# Ureteric stenting vs not stenting following uncomplicated ureteroscopic lithotripsy: A prospective randomised trial

**DOI:** 10.1080/2090598X.2020.1762280

**Published:** 2020-05-19

**Authors:** Saddam Al Demour, Adel Alrabadi, Abedallatif AlSharif, Mera Ababneh, Raed Al-Taher, Motaz Melhem, Hammam Mansi, Sa’id Aljamal, Mohammad Abufaraj

**Affiliations:** a Department of Special Surgery, Division of Urology, The University of Jordan, School of Medicine, Amman, Jordan; b Department of Diagnostic Radiology and Nuclear Medicine, The University of Jordan, School of Medicine, Amman, Jordan; c Department of Clinical Pharmacy, Faculty of Pharmacy, Jordan University of Science and Technology, Irbid, Jordan; d Division of Pediatric Surgery, Department of General Surgery, University of Jordan, School of Medicine, Amman, Jordan

**Keywords:** Ureteroscopy, lithotripsy, uncomplicated, ureteric stent, ureteric stone

## Abstract

**Objective:**

To compare three groups of patients who underwent uncomplicated ureteroscopic lithotripsy (URSL) and to evaluate whether stenting could be eliminated after the procedure, as there is no consensus about whether a ureteric stent should be placed after uncomplicated ureteroscopy for stone retrieval.

**Patients and methods:**

In this randomised clinical trial (NCT04145063) 105 patients underwent uncomplicated URSL for ureteric stones. They were prospectively randomised into three groups: Group 1 (34 patients) with a double pigtail ureteric stent, Group 2 (35 patients) with a double pigtail ureteric stent with extraction string, and Group 3 (36 patients) with no ureteric stent placed after the procedure. The outcomes measured were: postoperative visual analogue scale (VAS) score for flank pain and dysuria score, urgency, frequency, suprapubic pain, haematuria, analgesia requirement, operative time, re-hospitalisation, and return to normal physical activity.

**Results:**

The mean (SD) operative time was significantly longer in groups 1 and 2 compared to Group 3, at 22.2 (9.1), 20.2 (6) and 15.1 (7.1) min, respectively (*P* < 0.001). The results of the VAS for flank pain and dysuria scores, urgency, frequency, haematuria, and suprapubic pain showed a significant difference at all time-points of follow-up, being significantly higher in groups 1 and 2 compared to Group 3 (all *P* < 0.001). Further analysis showed that measured outcomes, and analgesia need for groups 1 and 2 were similar, at all time-points except at week 1 and 1 month where Group 2 patients’ had less symptoms (*P* < 0.001).

**Conclusion:**

Double pigtail ureteric stent placement appears to be unnecessary in procedures considered ‘uncomplicated’ by operating urologists during surgery. The advantages of the double pigtail ureteric stent with extraction string over the double pigtail ureteric stent only include earlier and easier removal with earlier relief of symptoms, and less analgesia requirements.

**Abbreviations:**

KUB: plain abdominal radiograph of the kidneys, ureters and bladder; URSL: ureteroscopic lithotripsy; VAS: visual analogue scale

## Introduction

Urolithiasis is a major clinical and economic burden for healthcare systems; it is a highly prevalent condition with a high rate of recurrence and a substantial impact on quality of life [1,2]. The incidence and prevalence of stone disease are increasing, most likely due to changes in nutritional and environmental factors [3–5].

In the last decade, surgical management of ureteric stones has changed fundamentally due to improvements in instruments such as smaller calibre semi-rigid and flexible ureteroscopes and intracorporeal lithotripsy with laser energy. These advances have made ureteroscopy an outpatient procedure, less traumatic, safer and more effective for the treatment of stones in all locations of the ureter [6–8].

Ureteric stent insertion after ureteroscopic lithotripsy (URSL) is common practice and widely accepted as best practice for patients who are pregnant or have a solitary kidney, transplanted kidney or renal impairment [9]. Ureteric stenting is certainly necessary in complicated ureteroscopies involving bleeding, ureteric trauma, or large residual stone burden [10].

However, there is no consensus about whether a ureteric stent should be placed after uncomplicated ureteroscopy for stone retrieval. Furthermore, the definition of uncomplicated URSL remains controversial [11]. Despite this controversy, most urologists routinely insert ureteric stents, justified by the hypothetical fact that stent placement promotes the passage of residual stone fragments and clots, presumably lowers the risk of stricture formation, prevents ureteric obstruction and renal colic resulting from ureteric oedema following stone retrieval [12,13].

However, ureteric stent insertion after ureteroscopy is potentially associated with some morbidity including pain, infection and irritative voiding symptoms. Ureteric stent insertion may also result in more serious complications such as upward stent migration, sepsis, ‘forgotten stents’, or encrustation with stone formation, thereby increasing morbidity and costs [14–16]. The frequency and severity of these complications may be reduced by use of a double pigtail ureteric stent with an extraction string attached allowing for fast non-invasive stent removal [17]. Randomised prospective trials have found that routine stenting after uncomplicated ureteroscopy is not necessary because stenting might be associated with higher morbidity [18,19].

Comparative studies between these three approaches (double pigtail ureteric stent, double pigtail ureteric stent with extraction string, and no double pigtail ureteric stent insertion) are lacking; most of the available literature compares stenting with not stenting. We found no studies that compared all three approaches. In addition, amongst these studies, there was no consensus on the definition of uncomplicated URSL. Indeed, available literature evaluating the benefits of ureteric stenting often includes both uncomplicated and complicated ureteroscopy. This results in inhomogeneous groups of patients and does not allow the drawing of definitive conclusions.

In our present randomised prospective trial, we included simple visual criteria for defining uncomplicated URSL. A homogenous group of patients undergoing uncomplicated ureteroscopies for stone removal were randomised into three groups: double pigtail ureteric stent, double pigtail ureteric stent with extraction string, and no double pigtail ureteric stent.

## Patients and methods

### Study design and approval

This was a randomised controlled trial conducted between February 2016 and January 2019 at Jordan University Hospital in Amman, Jordan. The study protocol was approved by the Institutional Review Board in Jordan University Hospital and registered on clinicalTrials.gov. (NCT04145063). All participants were informed about the study design and signed written informed consent, in accordance with the Declaration of Helsinki, was obtained from every patient. Random allocation was done using a balanced blocked random number list.

### Patient recruitment

A total of 123 patients with unilateral ureteric stones who underwent ureteroscopy with stone removal were randomised into three equal groups. Group 1, comprised patients in whom a double pigtail ureteric stent was inserted after stone removal; Group 2, comprised patients in whom a double pigtail ureteric stent with extraction string was inserted; and Group 3, included those in whom no ureteric stent was inserted after stone removal.

### Inclusion and exclusion criteria

Patients aged ≥18 years with unilateral ureteric stones managed by URSL were included in the study. Exclusion criteria included a stone size of >2 cm, bilateral ureteric stones, incomplete stone removal due to impacted stones, failed ureteroscopic access to the stone, and stone migration to the kidney. Pregnancy, active UTI, solitary kidney, ureteric stents placed preoperatively, severe mucosal injury or perforation, or suspected additional ureteric pathology such as ureteric stricture, urothelial carcinoma, or polyp were also exclusion criteria.

### Patient assessment

All patients were admitted to the hospital and assessed preoperatively by history and physical examination. Laboratory data collected included full blood counts; kidney function tests including serum creatinine, urea, sodium and potassium; urine analyses; and urine cultures. Stone size and location were assessed preoperatively by plain abdominal radiograph of the kidneys, ureters and bladder (KUB) and by non-enhanced CT. Upper ureteric stones were defined as those located above the superior border of the sacroiliac joint. Mid-ureteric stones were defined as those located between the superior and inferior borders of the sacroiliac joint, and distal ureteric stones as those located below the inferior border of the sacroiliac joint.

### Technique

All procedures were performed under general anaesthesia by the same experienced surgeon (S.A.). Intravenous antibiotics were given to all patients at the time of anaesthesia induction and maintained throughout the hospital stay. Patients were then switched to oral antibiotics for another 3 days.

URSL consisted of cystoscopy, insertion of a safety guidewire (0.097 cm [0.038 inch]) into the ureter under fluoroscopic guidance, passage of a semi-rigid ureteroscope (8.9 F; Richard Wolf GmbH, Knittlingen, Germany) into the ureter next to the guidewire without ureteric dilatation. Stones were extracted under vision using a Dormia stone basket (1.9 F, 90-cm length) and, if required, intracorporeal pneumatic lithoclast (EMS LithoClast, Bern, Switzerland) was used to fragment the stones. At the end of the ureteroscopy, the ureter was inspected to exclude the presence of residual stones or ureteric injury.

URSL was defined as uncomplicated if the stone was fragmented and extracted without ureteric injury, which included ureteric perforation or severe mucosal injury. This was assessed visually by the operating urologist (S.A.) during ureteroscopy. If obvious mucosal injury or ureteric perforation was present, the procedure was considered complicated. Therefore, a double pigtail ureteric stent was inserted, and the case was excluded from the study.

For those patients randomised to Group 1, a double pigtail ureteric stent was inserted via cystoscopy under fluoroscopic guidance. For patients in Group 2, a double pigtail ureteric stent was inserted in the same manner, but with an extraction string fixed to the external genitalia. For patients in Group 3, the ureter was left without a double pigtail ureteric stent. The double pigtail ureteric stents were Percuflex™ Plus from Boston Scientific (Marlborough, MA, USA; 4.8 F, 28-cm length).

Operating times were calculated starting with the insertion of the cystoscope until the final removal of all endoscopes. For patients in Group 1, the double pigtail ureteric stent was left *in situ* for 2 weeks, and then the patients were readmitted to day surgery for stent removal under general anaesthesia. For patients in Group 2, the double pigtail ureteric stent with extraction string was left in for 48 h, and then removed on or before discharge.

### Outcome measures and follow-up

All patients were evaluated at 5 h after the procedure and on the first and second postoperative days during inpatient treatment. Patients were also evaluated at 1 and 4 weeks, and 3 months in the outpatient clinic. The outcomes of interest were flank pain, dysuria, urgency, frequency, suprapubic pain, haematuria, need of analgesia, and readmission to the hospital.

Preoperative and postoperative flank pain and dysuria scores were assessed using a 10-cm visual analogue scale (VAS) from 0 to 10, in which 0–3 represented mild pain, 4–6 represented moderate pain, and 7–10 represented severe pain. Frequency, urgency, suprapubic pain, and haematuria were assessed using face-to-face direct questions. Flank pain score, dysuria score, frequency, urgency, suprapubic pain, haematuria, and need for analgesia were measured during the hospital stay and after discharge. Postoperative imaging, including KUB and renal ultrasonography, was performed at 1 week and at 3 months in the clinic.

### Statistical analysis

Continuous variables were presented as means ± standard deviations (SDs), while categorical variables were presented as numbers and percentages. For continuous variables, the Kruskal–Wallis *H*-test was used to compare variables among the three arms of the study and the Mann–Whitney *U*-test was used to compare variables within each pair of arm. The chi-square test was used to compare categorical variables. All tests were two-sided, and statistical significance was set at a *P* ≤ 0.05. The analysis was carried out using the Statistical Package for the Social Sciences (SPSS®), version 22 (SPSS Inc., IBM Corp., Armonk, NY, USA). The sample size was determined based on α level of 0.05, 90% power and expected difference between any study groups and controls are around 30% (pain score of ≥7 in study group and ≤4 in control group).

## Results

**Figure 1.**
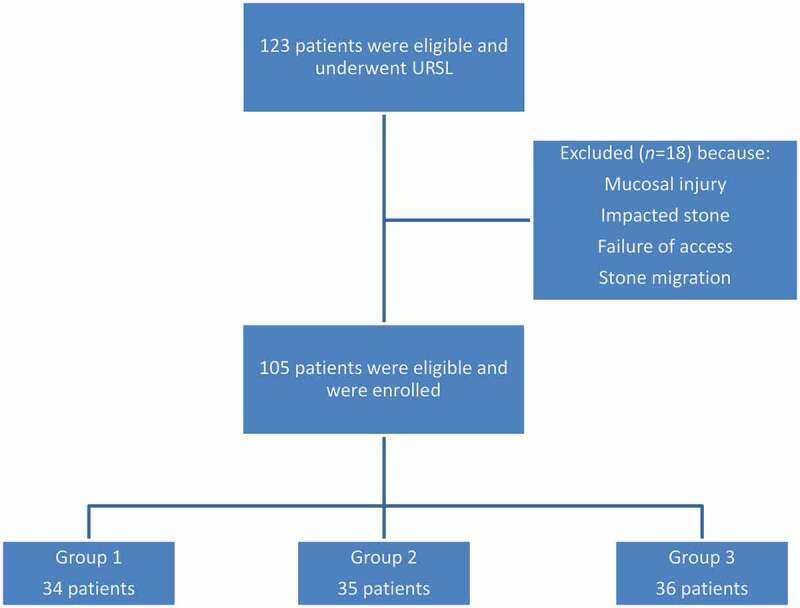
The flow of participants through the study.

A total of 123 patients were included. After URSL, we excluded 18 patients due to mucosal injury, impacted stones, failure of access, or stone migration. After that, Group 1 included 34, Group 2 included 35, and Group 3 included 36 patients (Figure 1).

The patients in the three study groups were comparable in their characteristics and methods of stone retrieval (Table 1). The mean operating time was statistically different among the groups (*P* < 0.001) (Table 1). All patients were discharged 2 days after the procedure. Two patients from Group 3 required re-hospitalisation 3 days after discharge due to severe flank pain with elevated serum creatinine; they were managed conservatively with antibiotics and analgesia. The stone-free rate was 100% at 3 months for all groups. We did not observe any case of hydronephrosis during follow-up.

**Table 1.** Patients demographic and clinical characteristics.

Variable	Group 1 Double pigtail ureteric stent	Group 2 Double pigtail ureteric stent with string	Group 3 No stent	P*
Number of patients	34	35	36	
Age, years, mean (SD)	46.2 (12.7)	40.8 (12.8)	43.8 (10.3)	0.2
Gender, *n* (%) Female Male	7 (20.6) 27 (79.4)	9 (25.7) 26 (74.3)	7 (19.4) 29 (80.6)	0.8
Education, *n* (%) Primary or secondary Bachelor or graduate	13 (38.2) 21 (61.8)	11 (31.4) 24 (68.6)	10 (27.8) 26 (72.2)	0.6
BMI, kg/m^2^, mean (SD)	27.8 (3.5)	27.1 (4.3)	28.8 (4.1)	0.2
Stone size, mm, mean (SD)	7.5 (2.4)	7 (1.5)	6.7 (1.6)	0.2
Stone location, *n* (%) Upper Middle Lower	12 (35.3) 19 (55.9) 3 (8.8)	16 (45.7) 14 (40) 5 (14.3)	14 (40) 17 (48.6) 4 (11.4)	0.8
Laterality, *n* (%) Right Left	16 (47.1) 18 (52.9)	15 (42.9) 20 (57.1)	23(63.9) 13(36.1)	0.2
Opacity, *n* (%) Opaque Lucent	7 (20.6) 27 (79.4)	7 (20) 28 (80)	4 (11.1) 32 (88.9)	0.5
Method of extraction, *n* (%) DB DB + PL	16 (47.1) 18 (52.9)	15 (42.9) 20 (57.1)	24 (66.7) 12 (33.3)	0.1
Operating time, min, mean (SD)	22.2 (9.1)	20.2 (6)	15.1 (7.1)	<0.001

BMI: body mass index; DB: Dormia basket; PL: pneumatic lithotripsy; * Kruskal–Wallis *H*-test for continuous data and chi-square test for categorical data.

The results of the VAS pain score for flank pain and dysuria showed a significant difference at all time-points of follow-up, with a higher mean VAS score in groups 1 and 2 compared to Group 3. Further analysis showed that scores for groups 1 and 2 were similar, except that Group 2 experienced less pain and dysuria at 1 week and at 1 month compared to Group 1 (Table 2).

**Table 2.** Score of flank pain and dysuria at different time points.

Variable	Group 1 Double pigtail ureteric stent (*n* = 34)	Group 2 Double pigtail ureteric stent with string (*n* = 35)	Group 3 No stent (*n* = 36)	*P*
Flank pain score (0–10*), mean (SD)				
At baseline	8.8 (1.5)	9.0 (1.6)	8.8 (1.5)	0.5^a^
				1 vs 3: 0.8^b^
				2 vs 3: 0.3^b^
				1 vs 2: 0.4^b^
At 5 h	5.4 (2.1)	5.8 (2)	3.1 (1.9)	<0.001
				1 vs 3: <0.001
				2 vs 3: <0.001
				1 vs 2: 0.5
At 24 h	4.8(2.1)	4.6 (2)	1.7 (2)	<0.001
				1 vs 3: <0.001
				2 vs 3: <0.001
				1 vs 2: 0.6
At 48 h	4.2 (1.9)	3.1 (1.8)	0.8 (1.1)	<0.001
				1 vs 3: <0.001
				2 vs 3 < 0.001
				1 vs 2 0.007
At day 7	4.6 (2.1)	0.9 (1.6)	0.6 (1.7)	<0.001
				1 vs 3: <0.001
				2 vs 3 0.4
				1 vs 2 0.001
At 1 month	1.4 (1.7)	0.14 (0.49)	0.02 (0.2)	<0.001
				1 vs 3 < 0.001
				2 vs 3 0.03
				1 vs 2 < 0.001
Dysuria score (0–10^a^), mean (SD)				
At 5 h	6.8 (1.9)	7.3 (1.8)	4.4 (2.5)	<0.001
				1 vs 3 0.001
				2 vs 3 < 0.001
				1 vs 2 0.4
At 24 h	6.3 (1.9)	6.4 (1.8)	2.1 (1.7)	<0.001
				1 vs 3 < 0.001
				2 vs 3 < 0.001
				1 vs 2 0.9
At 48 h	5.6 (1.5)	4.7 (1.6)	1.0 (1.1)	<0.001
				1 vs 3 < 0.001
				2 vs 3 < 0.001
				1 vs 2 0.3
At 7 days	5.6 (1.7)	1.7 (2)	0.4 (1.1)	<0.001
				1 vs 3 < 0.001
				2 vs 3 0.001
				1 vs 2 < 0.001
At 1 month	1.6 (1.7)	0.09 (0.4)	0.06 (0.3)	<0.001
				1 vs 3 0.001
				2 vs 3 0.6
				1 vs 2 < 0.001

*No pain (0) to extreme pain (10); ^a^: Kruskal–Wallis *H*-test; ^b^: Mann–Whitney *U*-test.

LUTS, namely, urgency, frequency, haematuria, and suprapubic pain, were less frequent in Group 3 compared to groups 1 and 2. Furthermore, these symptoms were significantly less frequent in Group 2 at 1 week and at 1 month compared to Group 1 (Table 3). There were statistically significant differences in the need for analgesia at all time-points of follow-up, with groups 1 and 2 requiring more analgesia than patients in Group 3 (Table 3).

**Table 3.** The presence of urgency, frequency, suprapubic pain, haematuria and need for analgesia.

	Time	Group 1 Double pigtail ureteric stent, *n* (%)	Group 2 Double pigtail ureteric stent with string, *n* (%)	Group 3 No stent, *n* (%)	P*
Urgency	5 h	32 (94.1)	35 (100)	21 (58.3)	<0.001
	24 h	30 (88.2)	34 (97.1)	7 (19.4)	<0.001
	48 h	30 (88.2)	31 (88.6)	3 (8.3)	<0.001
	7 days	29 (85.3)	9 (25.7)	1 (2.8)	<0.001
	1 month	9 (26.5)	2 (5.7)	1 (2.8)	<0.004
Frequency	5 h	32 (94.1)	35 (100)	21(58.3)	<0.001
	24 h	30 (88.2)	33 (94.3)	7 (19.4)	<0.001
	48 h	30 (88.2)	30 (85.7)	2 (5.5)	<0.001
	7 days	29 (85.3)	8 (22.9)	1 (2.8)	<0.001
	1 month	9 (26.5)	0	0	<0.001
Suprapubic pain	5 h	32 (94.1)	33 (94.3)	15 (41.7)	<0.001
	24 h	29 (85.3)	32 (91.4)	4 (11.1)	<0.001
	48 h	26 (76.5)	22 (62.9)	2 (5.5)	<0.001
	7 days	27(79.4)	6 (17.1)	1 (2.8)	<0.001
	1 month	5 (14.7)	1 (2.9)	0	0.01
Haematuria	5 h	29 (85.3)	34 (97.1)	26 (72.2)	0.008
	24 h	25 (73.5)	21 (60)	10 (27.9)	<0.001
	48 h	22 (64.7)	12 (34.3)	1 (2.8)	<0.001
	7 days	22 (64.7)	3 (8.6)	1 (2.8)	<0.001-
	1 month	1(2.94)	0	0	
Analgesia	5 h	31 (91.2)	34 (97.1)	21 (58.3)	<0.001
	24 h	28 (82,4)	29 (82.9)	8 (22.2)	<0.001
	48 h	24 (70.6)	17 (48.6)	3 (8.3)	<0.001
	7 days	26 (76.5)	8 (22.9)	1 (2.8)	<0.001

*Chi-square test.

Within 7 days of the procedure, 26 patients (91.7%) in Group 1, 33 (94.3%) in Group 2, and 35 (97.2%) in Group 3 returned to normal physical activity, and, within 1 month, all 105 patients returned to normal physical activity.

## Discussion

Ureteric stent placement after URSL is common clinical practice. It is traditionally encouraged to prevent complications, such as ureteric stricture, flank pain and renal failure due to ureteric oedema or passage of stone fragments and clots [20]. On the other hand, the insertion of a ureteric stent after URSL may result in symptoms such as flank pain, urgency, dysuria, haematuria or other rare complications such as stent migration or urosepsis. In addition, routine ureteric stent placement may be associated with prolonged operating time and costs [17,21–23].

Ureteroscopy and intracorporeal lithotripsy are becoming less invasive, resulting in decreased morbidity. Postoperative stenting has become the most frequent cause of morbidity in ureteroscopic stone removal. Joshi et al. [24] and Ucuzal et al. [25] reported that patients with stents had undesirable consequences and a significantly negative impact on patients’ quality of life. Therefore, whether it is necessary to routinely insert a stent after ureteroscopy for ureteric calculi remains controversial.

This ongoing controversy has prompted the use of stents with extraction strings to prevent early complications of the stone removal, particularly ureteric obstruction. This would also allow non-invasive stent removal and reduction in stent dwell time, ultimately leading to decreased morbidity, physical burden, and overall procedural costs [26,27].

Comparative studies between stent, no stent, and stent with an extraction string in patients who underwent uncomplicated URSL are lacking. The definition of uncomplicated URSL used in our present study is simple and based principally on visual assessment by the operating urologist. The results of the present study cannot be generalised to patients undergoing complicated URSL, as they were excluded.

In our present prospective, randomised controlled study, flank pain scores were lowest for patients in Group 3 at all time-points. Flank pain scores for patients in Group 2 dropped to levels comparable to those in Group 3 by the end of the first week. These scores were significantly lower than those in Group 1. In our present study, the pain score results in stented and unstented groups were in agreement with some previous studies, although some studies reported contradictory results [28,29].

These discrepant results may be due to the use of different methods for pain assessment; few studies used categorical assessments of pain (mild, moderate, or severe), while others used a more detailed scale (10-cm VAS). In addition, time intervals for pain assessment varied among these studies.

Similarly, dysuria scores were the lowest for patients in Group 3 at all time-points. Dysuria for patients in groups 1 and 2 were comparable in the first 48 h. However, by the end of first week, dysuria scores for patients in Group 2 dropped significantly in comparison to those in Group 1. At 1 month, patients in groups 2 and 3 had comparable dysuria scores and still reported significantly less dysuria than patients in Group 1.

As expected, patients in Group 3 reported less urgency, frequency, suprapubic pain, and haematuria compared to those in groups 1 and 2 at all time-points. The presence of these symptoms dropped significantly for patients in Group 2 after the stent with extraction string was removed. LUTS were likely related to the presence of the stent and irritation induced by presence of a foreign body [19]. Reduction in LUTS is the most significant advantage of the stent with a string approach. These findings are concordant with a study by Djaladat et al. [30], who used short-term ureteric stents attached to a Foley catheter, allowing early and easy stent removal.

Patients in Group 3 required less analgesia than patients in other groups at all time-points. It was also found that there was a sharp decline in analgesia requirements for Group 2 patients after stent removal (82.9% of patients required analgesia at 24 h, and only 22.9% at 7 days). Meanwhile, 76.5% of patients in Group 1 continued to require analgesia at 1 week.

The exact aetiology of symptoms related to ureteric stent insertion is unknown. It has been postulated that the intravesical portion of the stent results in increased pressure in the renal pelvis during micturition, as well as increased bladder mucosa irritation [31,32].

As shown in Table 3, the presence of these symptoms was observed more often in Group 2 patients for the first 48 h. We believe that foreign body irritation (i.e. the extraction string) adds to the LUTS.

The success of URSL is usually measured by the stone-free rate at follow-up. None of our patients was found to have any residual stone at 3 months, meaning that the success rate of URSL was identical in all three groups (100%).

Another advantage of an unstented approach is faster operating time (mean 15.1 min). Double pigtail ureteric stent with an extraction string had a similar operation time as double pigtail ureteric stent only, at a mean of 20.2 vs 22.2 min. However, patients with a stent with an extraction string did not require a second admission for stent removal; consequently, they experienced faster total operating times, less anaesthesia, and lower costs.

The only disadvantage found for the unstented approach was the readmission rate; two of our patients were re-hospitalised 3 days after the procedure for management of pain and an increased serum creatinine level. The two patients in Group 3 were managed conservatively without insertion of a ureteric sent and they were discharged after 3 days. However, this finding should not discourage urologist from not stenting patients after URSL, as the favourable outcomes of unstented groups were higher compared to patients with ureteric stents. None of the patients who had a stent with a string had any spontaneous stent dislodgment or urgent readmission.

The present study has several limitations. First, we have not provided a validated assessment of cost, quality of life and symptom score for the assessment of flank pain or LUTS such as the Ureteric Stent Symptom Questionnaire. Second, the type and amount of analgesic medications were not standardised across study arms during the follow-up affecting the interpretation of the results. Also, the stone-free status was determined mostly based on intraoperative visualisation rather than imaging studies, introducing potential biases. Nevertheless, and despite these limitations, our present data represent a real-life patient population that provide urologists with necessary information that may help in decision making and patient counselling.

## Conclusion

The unstented approach resulted in the smoothest postoperative course, and stent placement is not necessary in procedures determined to be uncomplicated by operating urologists at the time of surgery. The advantages of the double pigtail ureteric stent with extraction string over the double pigtail ureteric stent only included earlier relief of flank pain and dysuria, rapid relief of urgency, frequency, suprapubic pain, and haematuria, and less analgesia requirements. Furthermore, patients who had a stent with extraction string did not require a second procedure for stent removal, and none had spontaneous stent dislodgment.

## References

[1] Semins MJ, Matlaga BR. Medical evaluation and management of urolithiasis. Ther Adv Urol. 2010;2:3–9. 21789078 10.1177/1756287210369121 PMC3126068

[2] Scales CD Jr, Smith AC, Hanley JM, et al. Prevalence of kidney stones in the United States. Eur Urol. 2012;62:160–165. 22498635 10.1016/j.eururo.2012.03.052 PMC3362665

[3] Romero V, Akpinar H, Assimos DG. Kidney stones: a global picture of prevalence, incidence, and associated risk factors. Rev Urol. 2010;12:e86–96. 20811557 PMC2931286

[4] Prezioso D, Illiano E, Piccinocchi G, et al. Urolithiasis in Italy: an epidemiological study. Arch Ital Urol Androl. 2014;86:99–102. 25017588 10.4081/aiua.2014.2.99

[5] Seklehner S, Laudano MA, Jamzadeh A, et al. Trends and inequalities in the surgical management of ureteric calculi in the USA. BJU Int. 2014;113:476–483. 24053734 10.1111/bju.12372

[6] Ibrahim HM, Al-Kandari AM, Shaaban HS, et al. Role of ureteral stenting after uncomplicated ureteroscopy for distal ureteral stones: a randomized, controlled trial. J Urol. 2008;180:961–965. 18639269 10.1016/j.juro.2008.05.030

[7] Francesca F, Scattoni V, Nava L, et al. Failures and complications of transurethral ureteroscopy in 297 cases: conventional rigid instruments vs. small caliber semirigid ureteroscopes. Eur Urol. 1995;28:112–115. 8529733 10.1159/000475032

[8] Yaycioglu O, Guvel S, Kilinc F, et al. Results with 7.5 F versus 10 F rigid ureteroscopes in treatment of ureteral calculi. Urology. 2004;64:643–646. 15491688 10.1016/j.urology.2004.05.050

[9] Knudsen BE, Beiko DT, Denstedt JD. Stenting after ureteroscopy: pros and cons. Urol Clin North Am. 2004;31:173–180. 15040413 10.1016/S0094-0143(03)00091-0

[10] Foreman D, Plagakis S, Fuller AT. Should we routinely stent after ureteropyeloscopy? BJU Int. 2014;114:6–8. 25070223 10.1111/bju.12708

[11] Auge BK, Sarvis JA, L’Esperance JO, et al. Practice patterns of ureteral stenting after routine ureteroscopic stone surgery: a survey of practicing urologists. J Endourol. 2007;21:1287–1292. 18042016 10.1089/end.2007.0038

[12] Cevik I, Dillioglugil O, Akdas A, et al. Is stent placement necessary after uncomplicated ureteroscopy for removal of impacted ureteral stones? J Endourol. 2010;24:1263–1267. 20615145 10.1089/end.2009.0153

[13] Hosking DH, McColm SE, Smith WE. Is stenting following ureteroscopy for removal of distal ureteral calculi necessary? J Urol. 1999;161:48–50. 10037365

[14] Picozzi S, Carmignani L. A knotted ureteral stent: A case report and review of the literature. Urol Ann. 2010;2:80–82. 20882161 10.4103/0974-7796.65108 PMC2943687

[15] Schulze KA, Wettlaufer JN, Oldani G. Encrustation and stone formation: complication of indwelling ureteral stents. Urology. 1985;25:616–619. 4012953 10.1016/0090-4295(85)90293-6

[16] Rembrink K, Goepel M, Meyer-Schwickerath M. The forgotten double J stent. Urol Int. 1992;49:119–120. 1441012 10.1159/000282406

[17] Joshi HB, Stainthorpe A, MacDonagh RP, et al. Indwelling ureteral stents: evaluation of symptoms, quality of life and utility. J Urol. 2003;169:1065–1069. 12576847 10.1097/01.ju.0000048980.33855.90

[18] Pengfei S, Yutao L, Jie Y, et al. The results of ureteral stenting after ureteroscopic lithotripsy for ureteral calculi: a systematic review and meta-analysis. J Urol. 2011;186:1904–1909. 21944085 10.1016/j.juro.2011.06.066

[19] Song T, Liao B, Zheng S, et al. Meta-analysis of postoperatively stenting or not in patients underwent ureteroscopic lithotripsy. Urol Res. 2012;40:67–77. 21573923 10.1007/s00240-011-0385-7

[20] Harmon WJ, Sershon PD, Blute ML, et al. Ureteroscopy: current practice and long-term complications. J Urol. 1997;157:28–32. 8976208 10.1016/s0022-5347(01)65272-8

[21] Wang H, Man L, Li G, et al. Meta-analysis of stenting versus non-stenting for the treatment of ureteral stones. PloS One. 2017;12:e0167670. 28068364 10.1371/journal.pone.0167670 PMC5221881

[22] Nabi G, Cook J, N’dow J, et al. Outcomes of stenting after uncomplicated ureteroscopy: systematic review and meta-analysis. BMJ. 2007;334:572. 17311851 10.1136/bmj.39119.595081.55 PMC1828345

[23] Richter S, Ringel A, Shalev M, et al. The indwelling ureteric stent: a ‘friendly’procedure with unfriendly high morbidity. BJU Int. 2000;85:408–411. 10691815 10.1046/j.1464-410x.1998.00543.x-i1

[24] Joshi HB, Okeke A, Newns N, et al. Characterization of urinary symptoms in patients with ureteral stents. Urology. 2002;59:511–516. 11927301 10.1016/s0090-4295(01)01644-2

[25] Ucuzal M, Serçe P. Ureteral stents: impact on quality of life. Holist Nurs Pract. 2017;31:126–132. 28181978 10.1097/HNP.0000000000000200

[26] Oliver R, Wells H, Traxer O, et al. Ureteric stents on extraction strings: a systematic review of literature. Urolithiasis. 2018;46:129–136. 27324264 10.1007/s00240-016-0898-1 PMC5852195

[27] Barnes KT, Bing MT, Tracy CR. Do ureteric stent extraction strings affect stent‐related quality of life or complications after ureteroscopy for urolithiasis: a prospective randomised control trial. BJU Int. 2014;113:605–609. 24765679 10.1111/bju.12541

[28] El Harrech Y, Abakka N, El Anzaoui J, et al. Ureteral stenting after uncomplicated ureteroscopy for distal ureteral stones: a randomized, controlled trial. Minim Invasive Surg. 2014;2014:892890. 25431663 10.1155/2014/892890 PMC4241699

[29] Kenan I, Salih B, Suat E, et al. Is routine ureteral stenting necessary after uncomplicated ureteroscopic lithotripsy for lower ureteral stones larger than 1 cm? Urol Res. 2008;36:115–119. 18385992 10.1007/s00240-008-0135-7

[30] Djaladat H, Tajik P, Payandemehr P, et al. Ureteral catheterization in uncomplicated ureterolithotripsy: a randomized, controlled trial. Eur Urol. 2007;52:836–841. 17258387 10.1016/j.eururo.2007.01.042

[31] Thomas RA. Indwelling ureteral stents: impact of material and shape on patient comfort. J Endourol. 1993;7:137–140. 8518826 10.1089/end.1993.7.137

[32] Maldonado-Avila M, Garduño-Arteaga L, Jungfermann-Guzman R, et al. Efficacy of tamsulosin, oxybutynin, and their combination in the control of double-j stent-related lower urinary tract symptoms. Int Braz J Urol. 2016;42:487–493. 27286111 10.1590/S1677-5538.IBJU.2015.0186 PMC4920565

